# Modeling invasion of metastasizing cancer cells to bone marrow utilizing ecological principles

**DOI:** 10.1186/1742-4682-8-36

**Published:** 2011-10-03

**Authors:** Kun-Wan Chen, Kenneth J Pienta

**Affiliations:** 1 Department of Internal Medicine, The University of Michigan, 7308 CCC, 1500 E. Medical Center Drive, Ann Arbor, MI 48109, USA; 2 Department of Urology, The University of Michigan, 7308 CCC, 1500 E. Medical Center Drive, Ann Arbor, MI 48109, USA; 3 Department of Michigan Center for Translational Pathology, The University of Michigan, 7308 CCC, 1500 E. Medical Center Drive, Ann Arbor, MI 48109, USA

**Keywords:** ecosystem, cancer, population biology, metastasis, invasion, hematopoietic niche

## Abstract

**Background:**

The invasion of a new species into an established ecosystem can be directly compared to the steps involved in cancer metastasis. Cancer must grow in a primary site, extravasate and survive in the circulation to then intravasate into target organ (invasive species survival in transport). Cancer cells often lay dormant at their metastatic site for a long period of time (lag period for invasive species) before proliferating (invasive spread). Proliferation in the new site has an impact on the target organ microenvironment (ecological impact) and eventually the human host (biosphere impact).

**Results:**

Tilman has described mathematical equations for the competition between invasive species in a structured habitat. These equations were adapted to study the invasion of cancer cells into the bone marrow microenvironment as a structured habitat. A large proportion of solid tumor metastases are bone metastases, known to usurp hematopoietic stem cells (HSC) homing pathways to establish footholds in the bone marrow. This required accounting for the fact that this is the natural home of hematopoietic stem cells and that they already occupy this structured space. The adapted Tilman model of invasion dynamics is especially valuable for modeling the lag period or dormancy of cancer cells.

**Conclusions:**

The Tilman equations for modeling the invasion of two species into a defined space have been modified to study the invasion of cancer cells into the bone marrow microenvironment. These modified equations allow a more flexible way to model the space competition between the two cell species. The ability to model initial density, metastatic seeding into the bone marrow and growth once the cells are present, and movement of cells out of the bone marrow niche and apoptosis of cells are all aspects of the adapted equations. These equations are currently being applied to clinical data sets for verification and further refinement of the models.

## Background

A patient with prostate cancer, Mr. S. presents to clinic for consideration of further therapy. He is a 65 year old man that was diagnosed 12 years ago with a poorly differentiated, localized prostate cancer (PCa) when he presented for a routine physical exam and was found to have an elevated prostate specific antigen (PSA) blood test. Digital rectal exam revealed no abnormalities but prostate ultrasound and biopsy revealed a Gleason 5+4 = 9 cancer (clinical stage T1cNxMx). Because Mr. S. was in otherwise excellent health, he chose to undergo a radical retropubic prostatectomy and his prostate was removed. All of his lymph nodes were negative for cancer. He was considered to be cured of his disease.

Five years later, Mr. S's PSA became detectable and he now has 3 lesions present on bone scan. He has metastatic prostate cancer, now incurable. Each year, approximately 40,000 men who "should" have been cured of their prostate cancer by surgery or radiation therapy present with incurable metastatic disease that will manifest itself as metastatic lesions in the bone, usually years after primary treatment. The only explanation for this is that disseminated tumor cells (DTCs) are present in the bone microenvironment before surgery or radiation eradicated the primary tumor. How these cells traffic to the bone, become dormant, and then ultimately begin to proliferate are subjects of great interest to the cancer field.

Mr. S's cancer experience is not unique. PCa remains the most common cancer and the second leading cause of cancer-related death in American men today. Approximately 72% of patients who undergo radical prostatectomy had DTCs in their marrow prior to surgery suggesting that marrow dissemination is an early event in the progression of PCa disease. Clearly the ability of DTCs to proliferate, undergo apoptosis or become dormant must occur soon after the initial arrest of circulating tumor cells (CTCs) in the marrow. Unquestionably, a greater understanding of the molecular events that regulate a DTC's ability to become, and remain dormant over long periods is crucial to define new therapeutic strategies to combat disease progression. New mathematical models to understand these events may help further define strategies for understanding mechanisms of cancer cell trafficking and subsequent dormancy.

Eugene Odum, one of the founders of the science of ecology, defined an ecosystem as: "Any unit that includes all of the organisms (the biotic community) in a given area interacting with the physical environment so that a flow of energy leads to clearly defined biotic structures and cycling of materials between living and nonliving components is an ecological system" [1]. The ecosystem is the first unit of the ecological hierarchy that is complete, i.e., that has all of the necessary components for survival. Ecosystems do not exist independently, but interact in a complex web of relationships that connect all ecosystems to make up the biosphere [1]. Using the ecosystem paradigm, cancer cells, growing in an organ, can be considered to be a species co-existing in a complex habitat with other host cells. Together, the cancer cells and host cells, interacting within their habitat, create an ecosystem. This ecosystem, in turn, exists within a larger environment (the host patient as biosphere). The study of ecology, then, has the potential to offer insights into tumor biology. This is especially true for the study of metastasis [2,3].

Ecologists have studied the population biology of invasive species for decades and have documented their impact on local environments as well as the global ecosystem as a whole [4-6]. Invasive species start as a native population within a defined community and are then transported by some means to a new environment [4]. In this new environment, the invader either then dies off or enters a period of time during which it establishes itself (lag period). It then begins to spread and have impact on the local environment, disrupting the ecosystem as a whole. This disruption has broad implications for the native species and the broader ecosystem [4]. Biologic traits that result in a robust invasive species include rapid proliferative capacity, adaptation to environmental stress (phenotypic plasticity) and high tolerance to environmental heterogeneity [7-10].

**Figure 1:**
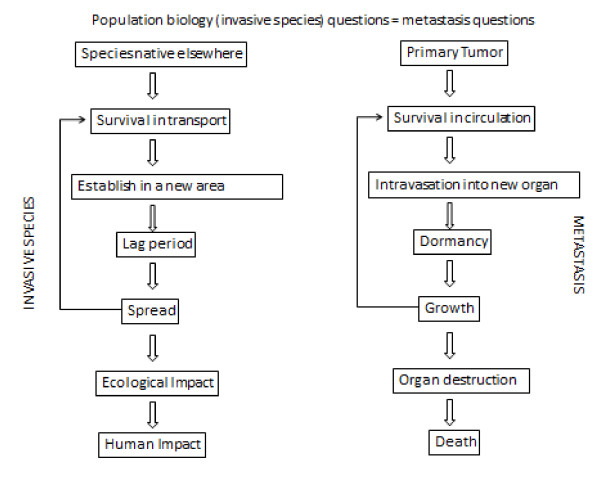
**Population biology of invasive species and metastasis**. The generalized steps necessary for a species to invade a new ecologic niche are directly analogous to the steps of cancer metastasis (modified from Sakai and colleagues [4]). The transport, establishment, and spread of invasive species can be compared to the intravasation of cancer cells into the blood stream where they are transported to a target organ where they extravasate. The cancer cells then enter a dormant period (lag period for invasive species) before growing, and displacing the host cells (native species). This results in damage to the local organ and eventually the host patient (human impact).

The life cycle of invasive species is directly analogous to the study of cancer metastasis [Figure 1] [4-10]. Cancer must grow in a primary site, extravasate and survive in the circulation to then intravasate at a target organ (invasive species survival in transport). Cancer cells often lay dormant at their metastatic site for a long period of time (lag period) before proliferating (invasive spread). Proliferation in the new site has an impact on the target organ microenvironment (ecological impact) and eventually the human host (biosphere impact) [11-13]. Studies of the population biology of invasive species have allowed a more precise focus on specific characteristics involved in invasiveness [14-16]. Tilman has described mathematical equations for the competition between species in a structured habitat [16]. These equations can be adapted to study the invasion of cancer cells into the bone marrow microenvironment as a structured habitat. Within the bone marrow microenvironment, hematopoietic stem cells (HSC) homing, quiescence, and self-renewal depend on the bone marrow HSC niche. A large proportion of solid tumor metastases are bone metastases, known to usurp HSC homing pathways to establish footholds in the bone marrow. Recent evidence suggests that tumor cells target and parasitize the HSC niche during metastasis just as invasive species do in the world [17]. We adapted the Tilman model of invasion dynamics to model cancer cell metastasis to the bone marrow microenvironment.

### Tilman's model of the dynamics of two species invasion into a structured habitat

If a single species cannot occupy all the sites in a habit, e.g., because of a certain death rate or natural movement in and out of the environment, another species may be able to invade into and survive in the open portion of a habitat. In Tilman's model [16], a superior species can displace the inferior species and occupy the lattice that was previously occupied by the inferior species. The inferior species, however, can colonize in the lattice only if the lattice of the fixed and homogeneous environment is neither occupied by superior species nor itself. The superior species occupies empty lattices with a constant colonization rate (birth rate), β_1_. The inferior species occupies empty lattices with a constant birth rate, β_2_. Either the death rate (μ_1_) of the superior species or the death rate of the inferior species (μ_2_) causes the occupied lattices to be emptied. It is assumed that the death rate and birth rate of the two species are independent of each other. Table 1 defines the symbols of the model.

**Table 1:** Symbols utilized in the equations

Symbol	Definition
***ρ***_**1**_	Density of species 1

***ρ***_**2**_	Density of species 2

***β***_**1**_	Birth rate of species 1

***β***_**2**_	Birth rate of species 2

***μ***_**1**_	Death rate of species 1

***β***_**2**_	Death rate of species 2

***κ***	The ability (proportion) that species 1 can displace species 2 by the growth (birth) of species 1

***v***	The ability (proportion) that species 2 can displace species 1 by the growth (birth) of species 2

***t***	Time

(1) $$\frac{\partial \rho_{1}}{\partial t}=\beta_{1}\rho_{1}\left(1-\rho_{1}\right)-\mu_{1}\rho_{1}$$

(2) $$\frac{\partial \rho_{z}}{\partial t}=\beta_{2}\rho_{2}\left(1-\rho_{1}-\rho_{2}\right)-\mu_{2}\rho_{2}-\beta_{1}\rho_{1}\rho_{2}$$

Eq. 1 describes that the dynamics of the superior species is only dependent on the colonization and its own death rate. Colonization, *β*_1 _*ρ*_1_(1-*ρ*_1_), indicates that when one member of the superior species grows, the member is not affected by any member of the inferior species. In other words, any member of superior species can displace the member of the inferior species. The part of the equation *μ*_1_*ρ*_1 _describes the density-independent mortality of superior species itself. Eq. 2 describes the dynamics of the inferior species is dependent on the colonization, mortality and the competitive displacement. Colonization, *β*_2_*ρ*_2_(1-*ρ*_1_-*ρ*_2_), indicates that the inferior species can colonize a lattice if it is unoccupied. A member of the inferior species, therefore, cannot displace any member of superior species. The competitive displacement, -*β*_2 _*ρ*_1 _*ρ*_2_, indicates that the inferior species is displaced by superior species growth (birth).

If Eq. 1 is set equal to zero, the equilibrium density for species 1 (superior species) can be calculated,

(3) $$\rho_{1}*=\left(1-\frac{\mu_{1}}{\beta_{1}}\right)$$

Since species 2 (inferior species) is affected by the species 1 (superior species), the equilibrium density for species 2 can be calculated after species 1 reach the equilibrium density. Similarly, if Eq. 2 is set equal to zero, the equilibrium density for species 2 (inferior species) can be calculated,

(4) $$\rho 2*=1-\frac{\mu_{2}}{\beta_{2}}-\rho_{1}*\left(1+\frac{\beta_{1}}{\beta_{2}}\right)=\frac{\mu_{1}}{\beta_{1}}-\frac{\mu_{2}}{\beta_{2}}+\frac{\mu_{1}}{\beta_{2}}-\frac{\beta_{1}}{\beta_{2}}$$

The initial density of the two different species does not affect the final equilibrium density. A very small initial density, however, requires more time to reach the equilibrium density.

### Adapting the invasion model to describe the invasion of cancer cells into the hematopoietic stem cell niche of the bone marrow microenvironment

**Figure 2:**
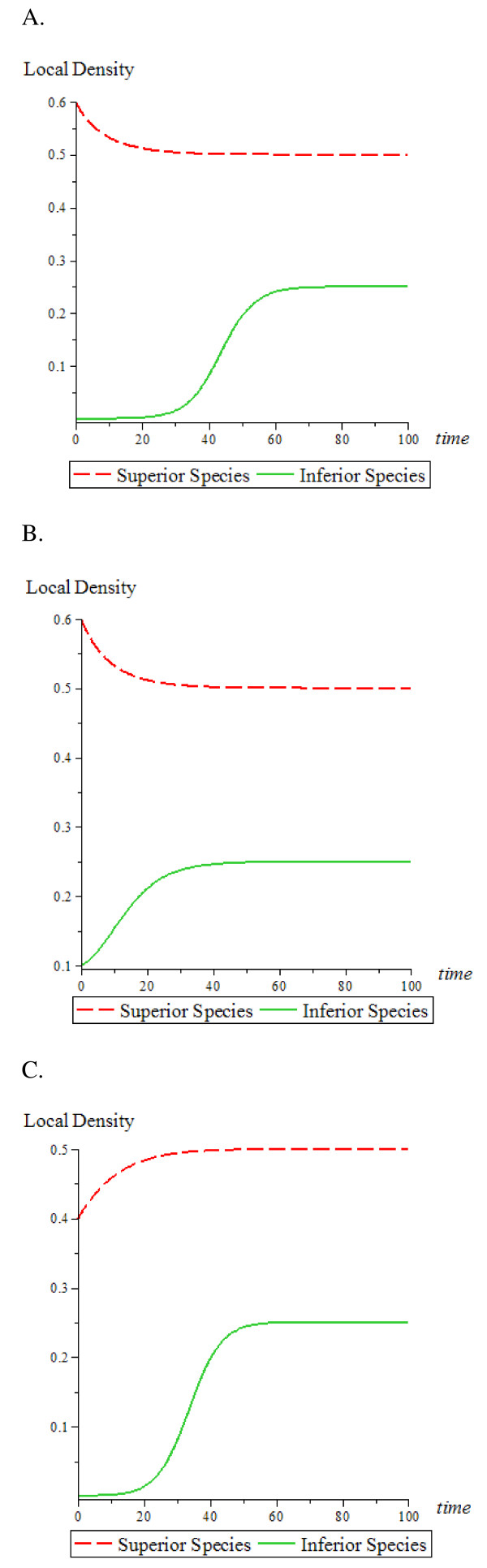
**Competition among two species: HSCs as the superior species**. The red (dash) line indicates the superior species (HSC); the green (solid) line indicates the inferior species (cancer cells). (A) superior species (HSC) has colonization rate *β*_1 _= 0.2, mortality rate *μ*_1 _= 0.1 *time*^-1^, and initial density = 0.6. The inferior species (cancer cells) has a colonization rate of *β*_2 _= 0.8, mortality rate *μ*_2 _= 0.1 *time*^-1^, and initial density = 0.0001. (B) The inferior species (cancer cells) has an initial density = 0.1; all other conditions remained the same as in (A). (C) Superior species (HSC) has an initial density = 0.4; all other conditions remained the same as in (A).

To better model cancer cell invasion, the Tilman equations were modified to take into account that HSCs are already present in the bone marrow and that cancer cells are the invading species. To observe competition between the two cell types, occupancy of a niche site can occur through colonization by movement into the site or by cell division (birth) rate. The avidity of the cell to occupy a niche site is reflected by a cell moving out of the niche or undergoing apoptosis (death rate). First, it was assumed that HSCs colonize the bone marrow niche in a more robust manner than cancer cells. i.e., HSCs are the superior species (Figure 2A, B and 2C). Under these conditions, colonization and niche occupancy of the superior species (HSC) is not affected by cancer cells. Furthermore, the initial density of either species does not affect the equilibrium density of both species, but does affect the time it takes to reach the equilibrium density. This may be a potential model for studying dormancy of cancer cells.

**Figure 3:**
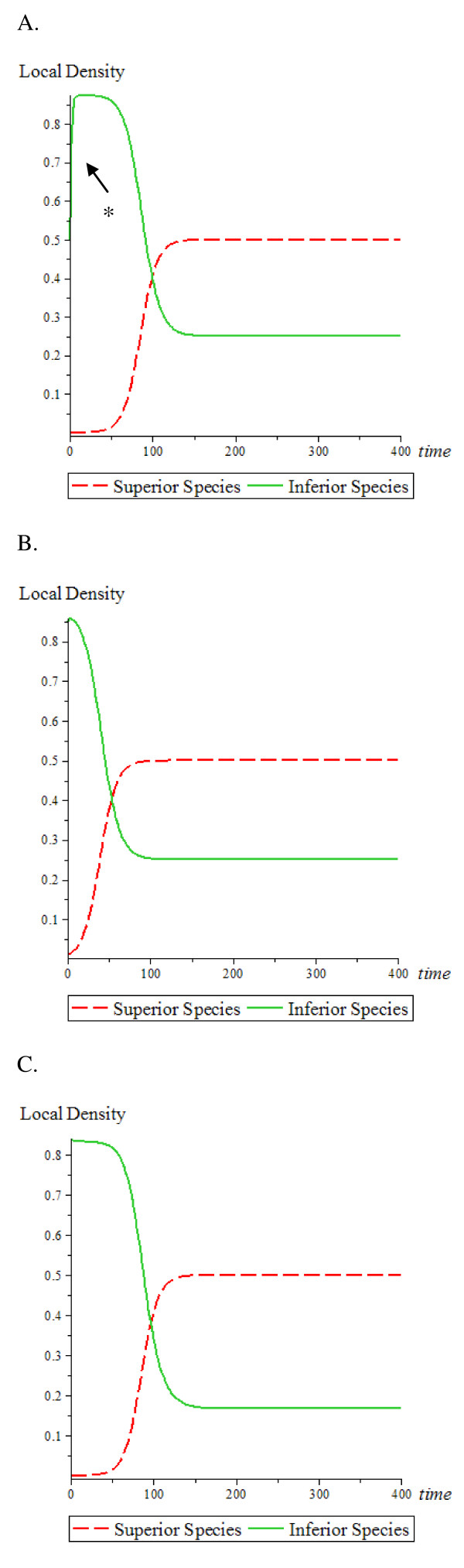
**Competition among two species: Cancer cells as the superior species**. The red (dash) line indicates the superior species (cancer cells); the green (solid) line indicates the inferior species (HSC). (A) Superior species (cancer cells) has a colonization rate of *β*_1 _= 0.2, mortality rate *μ*_1 _= 0.1 *time*^-1^, and initial density = 0.0001. Inferior species (HSC) has a colonization rate *β*_2 _= 0.8, mortality rate *μ*_2 _= 0.1 *time*^-1^, and initial density = 0.5. The * of the density curve of the inferior species is obtained from Eq. 2. (B) The superior species (cancer cells) has initial density = 0.01, and the inferior species (HSC) has initial density = 0.85; all other conditions remained the same as in (A). (C) The inferior species (HSC) has initial density = 0.84 and colonization rate *β*_2 _= 0.6 *time*^-1 ^; all other conditions remained the same as in (A).

Alternatively, it can be assumed that cancer cells are more avid for the bone marrow niche and are the superior species (Figure 3A, B and 3C). Notably, a high initial density is set for the inferior species (HSC) instead of the low initial density for the inferior species above. The observations found in Figure 2 were again demonstrated. First, although the superior species (cancer cells) has very low initial density, the superior species (cancer cells) is not affected by inferior HSC cells. Second, the initial density of either superior species (cancer cells) or inferior species (HSC) does not affect the equilibrium density of both species, but the initial density of the superior species (cancer cells) affects the time it takes to reach the equilibrium density. Third, compared to Figure 3(A) and 3(B), 3(C) shows a lower equilibrium density for the inferior species (HSC) because of a lower birth rate of species 2. The results are consistent with Eq. 4.

As noted, the Tilman's model describes the coexisting dynamics between superior and inferior species. However, the superior species is not affected by the inferior species. When modeling the real dynamics between HSC and cancer cells, it is doubtful that HSC or cancer cells behave in this absolute manner as a superior or inferior species. Thus, Tilman's model was amended to allow more interaction between the two species.

To take into account that both species have some ability to displace each other, the equations were further modified.

(5) $$\frac{\partial \rho_{1}}{\partial t}=\beta_{1}\rho_{1}\left[1-\rho_{1}-\left(1-k\right)\rho_{2}\right]-\mu_{1}\rho_{1}-\beta_{2}\rho_{2}\rho_{1}v$$

(6) $$\frac{\partial \rho_{z}}{\partial t}=\beta_{2}\rho_{2}\left[1-\left(1-v\right)\rho_{1}-\rho_{2}\right]-\mu_{2}\rho_{2}-\beta_{1}\rho_{1}\rho_{2}k$$

Coefficients, *k*(0 ≤ *k *≤ 1), expresses the ability (proportion) that species 1 can displace species 2 by the growth (birth) of species 1. Therefore, (1-*k*) expresses the proportion of species 2 that is not displaced by species 1. The colonization of the species 1, *β*_1_ρ_1_[1-ρ_1_-(1-k) ρ_2_], indicates that when the species 1 grows, the species 1 is affected by the (1-*k*) proportion of species 2 (Eq. 5). Species 1 can displace the *k *proportion of the species 2, but not all of species 2. Similarly, coefficients, *v *(0 ≤ *v *≤ 1), expresses the ability (proportion) that species 2 can displace species 1 by the growth (birth) of species 2. If *k *is larger than *v*, then species 1 has a stronger ability to displace species 2 (species 1 is the relative superior competitor).

The competitive displacement in Eq. 5, -*β*_2_*ρ*_2_*ρ*_1_*v*, indicates that the *v *proportion of species 1 is displaced by species 2's invasion or growth (birth in the equation). The competitive displacement in Eq. 6, -*β*_1_*ρ*_1_*ρ*_2_*k *, indicates that the *k *proportion of species 2 is displaced by species 1's birth. Both *k *and *v *express the displacement ability from species 1 and 2's birth, and are independent of each other. It is assumes that species 1 and species 2 do not change the lattice (occupancy of the niche) once they are given a birth until they are displaced. It is also assumes that when either species 1 or species 2 grow (birth rate), the lattice that they are going to occupy is chosen randomly. In addition, once either member of species 1 or species 2 is displaced, they will abandon the fixed and homogeneous space. Thus, the only reason that they may compete for the same lattice is because of the birth (movement in of a new cell or growth).

**Figure 4:**
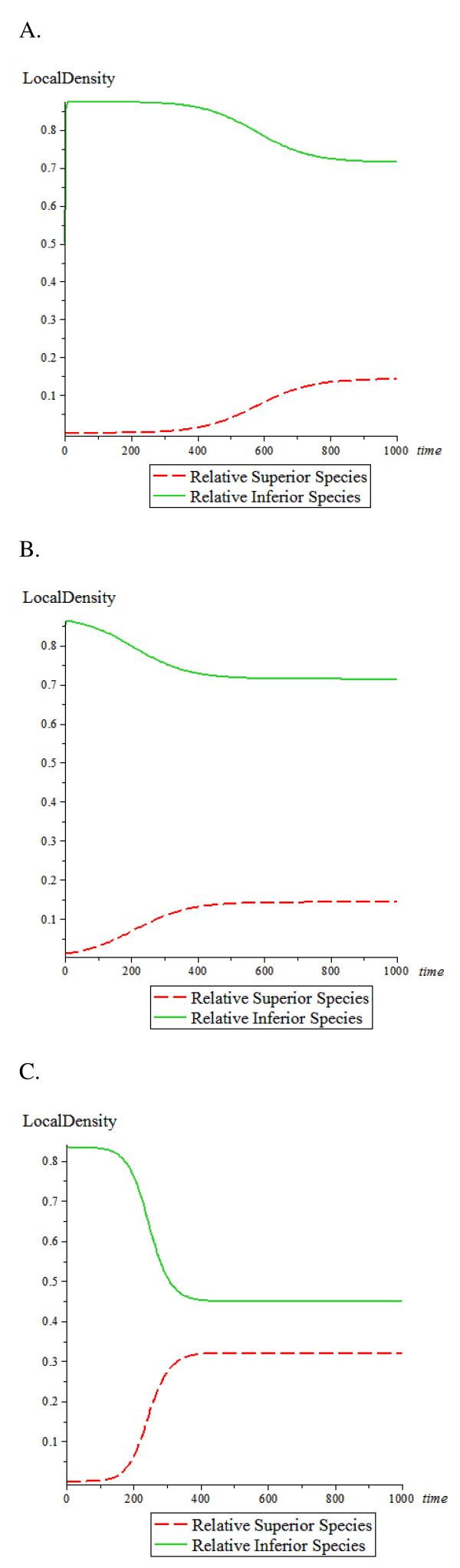
**Competition among two species**. The red (dash) line indicates the relative superior species (cancer cells); the green (solid) line indicates the relative inferior species (HSC). Superior species (cancer cells) can displace 90% of the inferior species (HSC). Inferior species (HSC) can displace 10% of the superior species (cancer cells). (A) Superior species (cancer cells) has colonization rate *β*_1 _= 0.2, mortality rate *μ*_1 _= 0.1 *time*^-1 ^, and initial density = 0.0001. Inferior species (HSC) has colonization rate *β*_2 _= 0.8, mortality rate *μ*_2 _= 0.1 *time*^-1 ^, and initial density = 0.5. (B) The superior species (cancer cells) has initial density = 0.01, and the inferior species (HSC) has initial density = 0.85; all other conditions remained the same as in (A). (C) The inferior species (HSC) has initial density = 0.84 and colonization rate *β*_1 _= 0.61 *time*^-1 ^; all other conditions remained the same as in (A).

Hematopoietic stem cells (HSC) and cancer cells were modeled as the two species. Resuming the scenario at Figure 3, it was assumed that cancer cells are the relative superior species and HSC are the relative inferior species (Figure 4A, B and 4C). To reflect what happens in cancer in the body, the initial density for the inferior species (HSC) was set at a high level and a low initial density was set for the invading cancer cells. All values that generated the curves in Figure 4A, B and 4C are equivalent to the values in Figure 3A, B and 3C, respectively. To demonstrate the simulations clearly, the time maximum was changed to 1000 in Figure 4. The equations applied to Figure 4 are:

$$\frac{\partial \rho_{1}}{\partial t}=\beta_{1}\rho_{1}\left[1-\rho_{1}-0.1\rho_{2}\right]-\mu_{1}\rho_{1}-\beta_{2}\rho_{2}\rho_{1}\times 0.1$$

$$\frac{\partial \rho_{2}}{\partial t}=\beta_{2}\rho_{2}\left[1-0.9\rho_{1}-\rho_{2}\right]-\mu_{2}\rho_{2}-\beta_{1}\rho_{1}\rho_{2}\times 0.9$$

κ=0.9,v=0.1

This results in cancer cells displacing most (90%) of the HSCs. However, the HSCs can also displace a small portion (10%) of the cancer cells. Comparing the conditions in Figure 3 versus Figure 4, the equilibrium densities of the cancer cells are lower in Figure 4 and it takes longer time for both species to reach their equilibrium densities in Figure 4.

## Conclusions

The Tilman equations for modeling the invasion of two species into a defined space have been modified to study the invasion of cancer cells into the bone marrow microenvironment. This required accounting for the fact that this is the natural home of hematopoietic stem cells and that they already occupy this structured space. These modified equations allow a more flexible way to model the space competition between the two cell species. The ability to model initial density, birth rate (metastatic seeding into the bone marrow and growth once the cells are present) and death rate (movement of cells out of the bone marrow niche and apoptosis of cells), are key components of this equations. The equations allow modeling of metastasis and the lag period of cancer cells. These equations are currently being applied to clinical data sets for verification and further refinement of the models. First, it is likely that during the progression from primary tumors to metastasis, genomic alterations occur, resulting in genetic and phenotypic changes. This article treated the cancer cells as a single species when in fact, it may be reasonable to treat them as multiple species based on their genetic heterogeneity (different genomes = different species) (18,19). The model can also be adapted to take into account mutation over time, i.e., the cancer cell population is relatively weak following initial metastasis, and becomes strong over time and dominates during the late stages. The fields of ecology and population biology are rich sources for understanding the biology of metastasis.

## Conflict of interest

The authors declare that they have no competing interests.

## Authors' contributions

KWC and KJP created the modeling. KWC developed the equations. KWC and KJP wrote the manuscript. Both authors have read and approved the final manuscript.
